# Sexual harassment from patient to medical student: a cross-sectional survey

**DOI:** 10.1186/s12909-022-03914-6

**Published:** 2022-11-30

**Authors:** Heather M. Mahurin, Jamie Garrett, Eliza Notaro, Vanessa Pascoe, Philip A. Stevenson, Katherine L. DeNiro, Michi M. Shinohara

**Affiliations:** 1 University of Washington School of Medicine, Seattle, Washington USA; 2 Department of Laboratory Medicine and Pathology, University of Washington, Seattle, Washington USA; 3 Division of Dermatology, Department of Medicine, University of Washington, 1959 NE Pacific St., Box 356524, Seattle, Washington 98195 USA; 4 Department of Dermatology, Beth Israel Deaconess Medical Center, Boston, MA USA; 5 Clinical Biostatistics, Fred Hutchinson Cancer Research Center, Seattle, Washington USA

**Keywords:** Sexual harassment, Sexual assault, Medical students, Burnout

## Abstract

**Background:**

There is little existing research investigating SH/SA specifically from patients to students. This study aims to assess the prevalence and impact of SH and SA from patient to medical student.

**Methods:**

A cross-sectional survey study was administered via electronic email list to all current medical students at the University of Washington School of Medicine (*n* = 1183) over a two-week period in 2019. The survey questions addressed respondents’ experiences with SH/SA from patients, frequency of reporting, and impact on feelings of burnout.

**Results:**

Three hundred eleven responses were received for a response rate of 26%; 268 complete responses were included in the final analysis. Overall, 56% of respondents reported ever experiencing SH from a patient. SH from a patient was reported by significantly more of those who identify as female compared to male (66% vs 31%; *p* < .001). Similar frequency of experiencing SH within the last year were reported by females and males (90% vs 88%; *p* = .96). Clinical students were more likely to have ever experienced SH compared to preclinical students (61% vs 39%; *p* < .001). The majority (86%) of respondents who experienced SH/SA did not report it in an official capacity. Those who identify as female were more likely to report that SH from a patient contributed to feelings of burnout (21% vs 5% for male; *p* = .02). Behaviors consistent with SA were experienced by 16% of respondents, with similar frequency between females and males.

**Conclusions:**

This study demonstrates that patient to medical student SH/SA is a common occurrence, particularly among students identifying as female. It also highlights the significant impact of SH/SA incidents on feelings of burnout.

**Supplementary Information:**

The online version contains supplementary material available at 10.1186/s12909-022-03914-6.

## Background

Sexual harassment (SH), according to the National Women’s Law Center, includes unwelcome sexual advances, requests for sexual favors, and/or hostile verbal or physical conduct that targets someone based on gender, whether sexual overtures are involved [1]. Between 2010 and 2019, charges of sex-based harassment filed to the United States Equal Employment Opportunity Commission exceeded 12,000 per year [2]. Sexual assault (SA) specifically refers to sexual contact or behavior, often physical, that occurs without the consent of the victim. SH and SA occurring in the workplace has been well researched, with most focus on SH from employers and between coworkers.

Medical students are not immune to experiencing SH during their educational training. A 2018 report from the National Academies of Sciences, Engineering, and Medicine (NASEM) showed female medical students were 220% more likely than non-science, technology, engineering, and math majors to experience SH by faculty/staff [3]. SH is frequently reported by medical students, with 70% of students reporting some type of harassment, most frequently by strangers [4] or patients [4–9]. SH of medical students can take the form of sexually explicit comments, inappropriate touching, unwelcome sexual advances, and humiliation [10, 11]. In a review of literature from 2014 to 2019, multiple journal articles [4–6, 8, 12–19] acknowledged SH affecting medical students, with most of these studies primarily addressing SH from supervisors, faculty, and other students.

There is little existing research, however, addressing SH specifically from patients to medical students. Medical students may be particularly vulnerable to SH from patients as it may be intended to reduce the student’s status, thereby increasing the patient’s feeling of control [4]. We hypothesized that medical students, particularly those who identify as female, frequently experience SH from patients which in turn has a profound impact on their psychological and physical well-being. In addition, we hypothesized that medical students rarely report SH/SA incidences when they do occur.

## Methods

An anonymous electronic survey was administered via electronic mailing list to all medical students enrolled at the University of Washington School of Medicine (*n* = 1183) over a two-week period during April 2019. Responses were collected using REDCap electronic data capture tools hosted at the University of Washington [20, 21]. This study was reviewed by the University of Washington Human Subjects Division and determined to be IRB exempt (#STUDY00005548). Statistical analysis was performed using STATA version 14.0 (StataCorp LP, College Station, TX) and R version 3.6.1 (R Foundation for Statistical Computing, Vienna, Austria). Logistic regression was used to compare responses between groups. Respondents with missing values were excluded from individual comparisons requiring those variables. *P* values < 0.05 were considered statistically significant.

Survey questions addressed demographics, respondents’ experiences with SH or SA from patient(s), and their responses to the SH or SA. SH was defined as per the United States Equal Employment Opportunity Commission definition as including “unwelcome sexual advances, requests for sexual favors, and other verbal or physical conduct of a sexual nature”. SA was defined as any of the following behaviors: unwanted, intentional, exposure of patient genitals; unwanted exposure to pornography or sexual content; unwanted, intentional touching of provider’s genitals, groin, or breasts.

## Results

### Survey respondents

A total of 311 survey responses were collected, for a response rate of 26%; 43 incomplete responses were excluded, leaving 268 respondents for the analysis. One hundred eighty-three respondents identified as female, 80 identified as male, 3 either did not identify as female/male, and 2 did not provide their sex. Given the small number of non-binary/not identified students, this group was excluded from comparative statistical analysis. The demographics of respondents are summarized in Table 1.

**Table 1:** Respondent characteristics (*N* = 268)

		Sex^**a**^	
	All (***N*** = 268)	Female (***N*** = 183)	Male (***N*** = 80)
	N (%)	N (%)	N (%)
Age			
< 24 years	31 (11.6)	21 (11.5)	10 (12.5)
25–29 years	181 (67.5)	128 (69.9)	50 (62.5)
30–34 years	41 (15.3)	26 (14.2)	13 (16.3)
35–44 years	15 (5.6)	8 (4.4)	7 (8.8)
≥ 45 years	0 (0)	0 (0)	0 (0)
Year in medical school			
First year	84 (31.3)	56 (30.6)	26 (32.5)
Second year	28 (10.4)	22 (12.0)	5 (6.3)
Third year	68 (25.4)	46 (25.1)	21 (26.3)
Fourth year	86 (32.1)	58 (31.7)	27 (33.8)
> Fourth year	2 (0.7)	1 (0.5)	1 (1.3)

^a^ Five respondents who did not identify as male or female were excluded from this analysis

### Frequency of SH

Overall, 56% (149/268) of respondents reported ever experiencing SH from a patient and 49% (131/268) reported experiencing SH from a patient in the last year. Of those who had incidents of SH, 51% described behaviors consistent with SA. Of those who experienced SH from a patient in the last year (*n* = 131), 91 (69%) reported experiencing SH 1–3 times over the year and 7 (5%) reported experiencing SH more than once a month.

SH from a patient was reported by significantly more females compared to males at any time (66% (121/183) vs 31% (25/80); *p* < .001) but similar frequencies of experiencing SH within the last year were reported (90% (106/118) of females and 88% (22/25) of males; *p* = .96). Of students who reported their gender as “other” (*n* = 3), two reported experiencing SH, while one was unsure. The most frequently experienced type of SH among the cohort overall was comments on the student’s appearance, with 53% of all respondents reporting this behavior (Table 2).

**Table 2:** Frequency and types of SH/SA behaviors experienced

		Sex^**a**^		
	All (***N*** = 268)	Female (***N*** = 183)	Male (***N*** = 80)	OR (CI)^**b**^
	N (%)	N (%)	N (%)	
Ever experienced SH	149 (55.6)	121 (66.1)	25 (31.3)	4.28 (2.45,7.48)
Experienced any of these behaviors:				
Comments on your appearance	142 (53.0)	116 (63.4)	23 (28.8)	4.29 (2.43,7.59)
Asked about marital or relationship status	95 (35.4)	78 (42.6)	17 (21.3)	2.75 (1.49,5.07)
Asked on a date	24 (9.0)	21 (11.5)	3 (3.8)	3.33 (0.96,11.49)
Told jokes or stories of a sexual nature	98 (36.6)	82 (44.8)	14 (17.5)	3.83 (2.01,7.30)
Other	32 (11.9)	22 (12.0)	9 (11.3)	1.08 (0.47,2.46)
Any of the above	148 (55.2)	120 (65.6)	25 (31.3)	4.19 (2.41,7.45)
Experienced any of these unwanted behaviors:				
Intentional exposure to genitals	22 (8.2)	18 (9.8)	4 (5.0)	2.07 (0.68,6.33)
Exposure to pornography or sexual content	4 (1.5)	3 (1.6)	1 (1.3)	1.32 (0.13,12.85)
Intentional touching of your genitals, groin, breasts	14 (5.2)	9 (4.9)	5 (6.3)	0.78 (0.25,2.39)
Other	14 (5.2)	8 (4.4)	5 (6.3)	0.69 (0.22,2.16)
Any of the above	42 (15.7)	30 (16.4)	11 (13.8)	1.22 (0.60,2.70)

*Abbreviations*: *SH* Sexual harassment, *SA* Sexual assault, *OR* Odds ratio, *CI* Confidence interval

^a^Five respondents who did not identify as male or female excluded from this analysis

^b^Odds ratio, females compared to males

In this cohort, 42% (112/268) of students were in the preclinical phase of medical school and 58% (156/268) were in the clinical phase. We found no significant difference in the proportion who reported experiencing SH in the past year when comparing clinical and preclinical students (90% (89/99) vs 85% (41/48); *p* = .43). However, clinical students were significantly more likely to have ever experienced SH ever when compared to preclinical students (61% (94/155) vs 39% (44/112); *p* < .001). Students were also asked in which practice settings they experienced the most frequent SH from patients, with outpatient private practice clinics (25%) and the inpatient setting of academic hospitals (17%) most commonly reported.

### Reporting SH incidents

When asked if they knew how to report unwanted sexual behavior from a patient, just over half (57%, 152/268) of all respondents indicated “probably yes” or “definitely yes”. Those who had experienced SH/SA (*n* = 149) were asked additional questions regarding reporting of the incident (Table 3). The majority (72%; 107/149) responded that they had experienced an incident of unwanted sexual behavior from a patient and did not report it in an official capacity, with no significant difference based on sex (*p* = .93). The most common reason overall why respondents didn’t report an incident of SH was “not sure if it was serious enough to report” (92%; 99/108). Those who identified as female were more likely to respond that they didn’t report an incident of SH/SA because they “weren’t sure it was serious enough”, they “did not think the patient intended to harass”, or they “did not think reporting would be productive” when compared to their male counterparts (Table 3).

**Table 3:** Reporting of SH/SA behaviors

		Sex^**a**^		
	All (***N*** = 149)	Female (***N*** = 183)	Male (***N*** = 80)	OR (CI)^**b**^
	N (%)	N (%)	N (%)	
Experienced SH/SA and did not report	107 (71.8)	89 (48.6)	18 (22.5)	1.06 (0.27,4.07)
Reasons for not reporting:				
Doubt or uncertainty about the incident				
Was not sure if serious enough	97 (90.6)	83 (77.6)	14 (13.1)	3.91 (2.05,7.46)
Did not think patient intended to harass	76 (71.0)	65 (60.7)	11 (10.3)	3.46 (1.71,6.99)
Was not sure it was SH/SA	52 (48.6)	43 (40.2)	9 (8.4)	2.42 (1.12,5.25)
Fear of retaliation				
Did not want anything to happen to the patient	35 (32.7)	29 (27.1)	6 (5.6)	2.32 (0.92,5.84)
Afraid of negative consequences from supervisors	28 (26.1)	23 (21.5)	5 (4.7)	2.15 (0.79,5.89)
Afraid of negative patient satisfaction	9 (8.4)	8 (7.5)	1 (0.9)	3.61 (0.44,29.37)
Feelings of shame, helplessness or hopelessness				
Felt helpless about what happened	13 (12.1)	11 (10.3)	2 (1.9)	2.49 (0.54,11.52)
Felt ashamed about what happened	12 (11.2)	9 (8.4)	3 (2.8)	1.33 (0.35,5.04)
Felt hopeless about what happened	6 (5.6)	6 (5.6)	0 (0)	n/a
Systems reasons				
Did not think reporting would be productive	84 (78.5)	71 (66.3)	13 (12.1)	3.27 (1.68,6.35)
Did not have time	36 (33.6)	34 (31.8)	2 (1.9)	8.90 (2.08,38.02)
Did not know how to report	28 (26.2)	24 (22.4)	4 (3.7)	2.86 (0.96,8.86)

*Abbreviations*: *SH* Sexual harassment, *SA* Sexual assault, *OR* Odds ratio, *CI* Confidence interval

^a^ Five respondents who did not identify as male or female excluded from this analysis

^b^Odds ratio, females compared to males

### Consequences of SH

Seventeen percent (35/208) reported that experiencing SH or SA by a patient contributed to feelings of burnout, with females 4.6 times more likely than males to report this (CI = 1.34,15.70). Six percent (9/149) of those who reported prior SH or SA reported having terminated a relationship with a patient based on the unwanted behavior. Nine students (6%) reported that they sought counseling or spoke with a mental health professional after experiencing unwelcome sexual behavior from a patient.

### Sexual assault

When asked if they had ever experienced behaviors consistent with SA, 16% (42/268) of respondents responded that they had experienced one or more behaviors, with similar rates between females and males (16% (30/183) vs 14% (11/80); Table 2). Of the 3 students who listed “other” as their gender, none reported experiencing SA behaviors.

## Discussion

Sexual harassment experienced by medical students can have a measurable impact on mental health, leading to burnout, anxiety, or even a career change [4, 22]. A 2014 meta-analysis which found that 59.4% of medical trainees had experienced at least one form of harassment or discrimination during their training, with more than two-thirds of the included studies reporting SH specifically [5]. Although much of the prior literature on SH focuses on harassment from faculty/staff, a Canadian national survey found that 40% of SH incidents were perpetrated by patients [8]. Our findings support that SH/SA by patients is a common experience encountered by medical students.

We found that SH behaviors are twice as likely to be experienced by students identifying as female, consistent with multiple previous studies that female students experienced SH two to three times more often than their male peers [4, 23, 24]. Similar frequency for experiencing SH (most commonly 1–3 times in the past year) was reported by both sexes, suggesting that though incidents are more likely to ever occur for females, incidents are infrequent in any given year. Our data demonstrate no significant difference between frequency of experiencing SA behaviors based on sex of the student. This could be due to the relatively small number of SA events reported in our cohort, but warrants further examination. Additionally, we found that students identifying as male are not immune from SH by patients, with 31% of male students having experienced SH. This prevalence is slightly higher than reported in prior studies which ranged from 12 to 29% [11, 25, 26]. The #MeToo movement has brought awareness of SH against females to the forefront. Greater dialogue and movement needs to occur to include men in the discussion, reducing the stigma associated with male sexual harassment and potentially increasing their reporting rates [27–29].

We hypothesized that medical students experience more SH compared to senior trainees (i.e., residents or fellows) and attending level physicians because of larger perceived power differential of hierarchical roles within academic medicine. However, we found the prevalence of SH among students in our cohort to be lower in comparison to our previous study of residents and attending physicians where we found that 83% reported experiencing SH and 31% SA behaviors from a patient [30]. This difference may be due to the greater number of cumulative patient encounters that residents and attendings have experienced. Our finding of a higher prevalence of experiencing SH from patients among clinical students compared to preclinical students likely also reflects increased time to accumulate SH incidents from patients, consistent with previous literature suggesting that SH and gender discrimination are higher in the clinical environment than in pre-clinical education [15, 31].

Since the inception of Title IX in 1972, medical schools and organizations have adopted a zero-tolerance policy on SH [12] including laws protecting victims from retaliation [13]. Despite this safety net, reporting of SH remains low. According to the Association of American Medical Colleges’ (AAMC) 2020 Medical School Graduation Questionnaire, only 26% of students who had experienced harassment or other offensive behaviors reported these incidents to faculty or medical school administrators. The most common reason for lack of reporting was that the incident did not seem important enough to report [32]. We confirmed that medical students who experienced SH from patients were unlikely to report SH in an official capacity.

In our cohort those who identified as female were more likely than their male counterparts to select reasons for not reporting that downplayed the episode, including “not sure if serious enough”, and “did not think the patient intended to harass”. In some environments, downplaying SH/SA may be a way for women who have experienced these incidents to transform them and lessen their impact [33]. Those who identify as female were also 3 fold more likely to report that they did not think reporting an incident would have productive consequences. This aligns with the concept that women perceive a greater risk in reporting SH than do men [4]. Some additional reasons students may fail to report include fear of reprimanding from attendings, humiliation from staff and peers, negative patient satisfaction or removal from a clinical rotation [13]. While we did find that fears of retaliation were also reported by our cohort as reasons for not reporting an incident of SH/SA, we did not see significant differences by sex. In order to ensure a safe learning environment, barriers to reporting must be lowered, there must be transparency about the outcomes of reporting, and safety and support must be ensured for all medical students who experience SH/SA.

One-fifth of our respondents noted that experiencing SH/SA by a patient made them feel burned out, with females reporting feeling burnout due to SH/SA more than males. Burnout has a higher prevalence among trainees and women in particular [34]. The connection between discrimination/harassment and poor mental health outcomes (e.g., depression, anxiety, burnout) has also been well-documented [35, 36], including in students who experience gender discrimination or harassment [22]. Men academic faculty members report burnout from experiencing direct harassment, whereas women report increased burnout with direct and indirect harassment experiences (i.e. witnessing or hearing about someone being directly harassed) [37]. The additional impact of indirect experiences of harassment may serve as an amplifier of the effects harassment and burnout for women in medicine.

### Limitations

There are several limitations to this study. The smaller sample size and survey population limited to a single public medical school may limit the generalizability of our findings. Due to the electronic methods utilized to distribute the survey, our data may be subject to selection and/or response bias. Another limitation that may cause bias is the relatively low response rate of 26%. Our sample had an overrepresentation of students identifying as female (68%) when compared to the percent of medical students who identify as female in the school overall (58%). The majority of our respondents were between the ages of 25–29 years, which is consistent with the national mean age of 24 years at time of matriculation to medical school [38], but narrower than the age range of matriculated students (21–37 years) in this school.

We did not specifically examine the prevalence and impact of SH/SA by race/ethnicity or among gender minority students. Existing literature about the rates of SH/SA experienced by people of different races/ethnicity is mixed, though data overall suggests some differences among groups. The Stop Street Harassment survey was a large (*n* = 2009) survey of adults that found no overall significant differences in the experience of SH/SA by race/ethnicity, although the authors did report a trend toward higher odds for SH for Hispanic women relative to white women [39]. In a study of Ob/Gyn residents, Hispanic/Latinx and white or Asian trainees reported higher incidences of sexual harassment compared to Black resident trainees [40]. Future studies are needed to investigate and address these potential race/ethnicity disparities of the experience of SH/SA. In addition, we did not perform analysis of non-binary students due to the small number of respondents identifying as such in our cohort. Given the high frequency of stigma and discrimination faced by transgender and gender non-binary medical students [41], further investigation of SH experienced by these students should be prioritized in future studies.

### Preventing and responding to SH/SA

It is clear that more needs to be done to reduce medical students’ exposure to SH/SA. Medical school leadership should prioritize the prevention and reduction of SH/SA. As suggested by Binder et al., one first step by medical schools is the public declaration of zero tolerance for harassment in addition to mandatory sexual harassment training programs [13]. Zero tolerance policies are especially important for those who supervise medical students. Allowing those in positions of power to commit or tolerate SH/SA could contribute to a culture of acceptance around SH/SA, including from patients, as work environments with a perceived organizational tolerance of SH tend to have higher overall rates of SH,(48) and SH is less likely to occur if sexually harassing behaviors are not accepted by authority figs [42]. Efforts to create diverse and inclusive environments may also help, as work environments which are male-dominated and with large power differentials between levels tend to have higher overall rates of SH [43–45].

Despite efforts and zero tolerance policies, it’s likely that we will never be able to prevent all SH/SA from patients. Inappropriate sexual behaviors can result from medical conditions, including delirium and dementia [46]. Given the likelihood of SH/SA occurring, it’s essential that those who supervise and/or work with medical students have formal training in bystander intervention [13]. Bystander intervention allows action be taken to call out and identify witnessed SH/SA behavior towards medical students, particularly if the witness is a supervisor or senior to the student (such as an attending or resident). Similar to microaggressions, allowing witnessed SH to occur without intervention or discussion implicitly suggests that the behavior is acceptable. Students should be educated on and empowered to report incidents, so that reporting is normalized, and the culture improves for future learners. Access to counseling and other support services and time off to utilize these services should also be readily available and easily accessible for affected students.

## Conclusions

We found that over half of medical students experienced SH from their patients during their training. Female medical students were twice as likely as their male counterparts to experience SH, and students were unlikely to report experiences of SH/SA regardless of their sex. As medical professionals we cannot tolerate SH/SA, regardless of whether it comes from a coworker or a patient. It is essential for students to feel supported and be aware of available resources if SH occurs. Addressing SH of medical students by patients will require medical schools and medical institutions to work together to provide a safe and effective learning environment.

## Supplementary Information

**Additional file 1.**

## Data Availability

The datasets used and/or analyzed during the current study are not publicly available due to requirements of the institutional review board, but are available from the corresponding author on reasonable request.

## Abbreviations

AAMC: Association of American Medical Colleges
NASEM: National Academies of Sciences, Engineering, and Medicine
SA: Sexual assault
SH: Sexual harassment
