# Supplementary material for: Sexual harassment from patient to medical student: a cross-sectional survey
Source: BMC Med Educ. 2022 Nov 30;22:824. doi: 10.1186/s12909-022-03914-6 (PMC9710121; doi:10.1186/s12909-022-03914-6)
Supplement: Supplementary file 1 — Additional file 1. [file 12909_2022_3914_MOESM1_ESM.pdf]

Patient to Medical Student Sexual Harassment in Medicine

The purpose of this survey is to investigate patient to medical student sexual harassment in medicine. Please complete this survey if you are a medical student (any year).

This survey should take < 10 min to complete. All survey responses are anonymous, and cannot be traced back to you.

This study was reviewed by the University of Washington Human Subjects Division and determined to be IRB exempt (#STUDY00005548).

Thank you for your participation.

Sincerely,

Jamie Garrett (E17), Katie Deniro MD, Eliza Notaro MD, Vanessa Pascoe MD, Michi Shinohara MD

University of Washington Dermatology & University of Washington School of Medicine

---

What gender do you identify with?

- ◯ Female
- ◯ Male
- ◯ Other
- ◯ Prefer not to say

Other, please explain:

____

What is your age?

- ◯ ≤24
- ◯ 25 - 29
- ◯ 30 - 34
- ◯ 35 - 44
- ◯ 45 - 54
- ◯ ≥55

What is your current year in medical school (excluding years spent in concurrent training programs, e.g. PhD, MPH)?

- ◯ First year
- ◯ Second year
- ◯ Third year
- ◯ Fourth year
- ◯ > Fourth year

SEXUAL HARASSMENT

According to the US Equal Employment Opportunity Commission, "sexual harassment" includes unwelcome sexual advances, requests for sexual favors, and other verbal or physical conduct of a sexual nature.

With this definition in mind, please answer the following questions about sexual harassment you may have experienced from patients at work or in training:

Do you feel that you've ever experienced sexual harassment from a patient?

- ◯ Definitely no
- ◯ Probably no
- ◯ Unsure
- ◯ Probably yes
- ◯ Definitely yes

How many times do you estimate you have experienced sexual harassment from a patient?

- ◯ Never
- ◯ 1-3 times
- ◯ 4-10 times
- ◯ 11-50 times
- ◯ >50 times

How frequently have you experienced sexual harassment from a patient in the past year?

- ◯ Never
- ◯ 1-3 times
- ◯ 4-10 times
- ◯ around once a month
- ◯ more than once a month

Which of the following behaviors have you experienced from a patient? (select all that apply)

- ☐ Comments on your appearance
- ☐ Asked about your marital or relationship status
- ☐ Asked on a date
- ☐ Told jokes or stories of a sexual nature
- ☐ Other
- ☐ None/does not apply

Other, please explain: ______________________________

If you have experienced sexual harassment from a patient, in which of the following practice settings did it occur? (select all that apply)

- ☐ Outpatient Academic Clinic
- ☐ Outpatient Veteran's Affairs (VA) Clinic
- ☐ Outpatient Private Practice Clinic
- ☐ Inpatient Academic Hospital
- ☐ Inpatient VA Hospital
- ☐ Inpatient Private Hospital
- ☐ Other
- ☐ None/does not apply

Other, please explain: ______________________________

Which of the following practice settings have you rotated through (or had other learning experiences) as a medical student? (select all that apply)

- ☐ Outpatient Academic Clinic
- ☐ Outpatient Veteran's Affairs (VA) Clinic
- ☐ Outpatient Private Practice Clinic
- ☐ Inpatient Academic Hospital
- ☐ Inpatient VA Hospital
- ☐ Inpatient Private Hospital
- ☐ Other
- ☐ None/does not apply

In which of the following practice settings have you experienced the most frequent sexual harassment from patients? (select one)	◯ Outpatient Academic Clinic<br>◯ Outpatient VA Clinic<br>◯ Outpatient Private Practice Clinic<br>◯ Inpatient Academic Hospital<br>◯ Inpatient VA Hospital<br>◯ Inpatient Private Hospital<br>◯ Other<br>◯ None/does not apply
Other, please explain:	__________________________________

Consider the following behaviors. Have you ever unwillingly experienced any of these behaviors from a patient?

☐ Unwanted, intentional, exposure of patient genitals
☐ Unwanted exposure to pornography or sexual content
☐ Unwanted, intentional touching of your genitals, groin, or breasts
☐ Other unwanted sexual behavior
☐ None of the above

Other, please explain: ______________________________

How frequently have you experienced any of these behaviors from a patient in the past year? (select one)

◯ Never
◯ 1-3 times
◯ 4-10 times
◯ around once a month
◯ more than once a month

Please answer the following questions about experienced unwelcome sexual advances, requests for sexual favors, and other verbal or physical conduct of a sexual nature or any other unwanted sexual behavior from a patient.

Have you ever reported an incident(s) of unwelcome sexual behavior from a patient to anyone at work (co-worker, attending, supervisor, administrator) in an official capacity?

- ◯ No
- ◯ Yes
- ◯ Unsure
- ◯ I have never experienced such an incident

Have you ever experienced an incident(s) of unwelcome sexual behavior from a patient and NOT reported it in an official capacity?

- ◯ No
- ◯ Yes
- ◯ Unsure
- ◯ I have never experienced such an incident

If you have ever NOT reported an incident(s), why not? (select all that apply):

- ☐ I wasn't sure it was actual sexual harassment/sexual assault.
- ☐ I didn't think patient intended to harass me.
- ☐ I wasn't sure if it was serious enough to report.
- ☐ I didn't have time.
- ☐ I didn't know how to report the incident.
- ☐ I didn't think reporting the incident would have productive consequences.
- ☐ I didn't want anything to happen to the patient.
- ☐ I was afraid of negative patient satisfaction.
- ☐ I was afraid of negative consequences from my supervisors at work.
- ☐ I felt ashamed.
- ☐ I felt helpless about what happened.
- ☐ I felt hopeless about what happened.
- ☐ Does not apply

Have you ever discussed an incident(s) of unwelcome sexual behavior from a patient with anyone (friend, family member, co-worker) in an UNofficial capacity?

- ◯ No
- ◯ Yes
- ◯ Unsure
- ◯ I have never experienced such an incident

Have you ever sought counseling or spoken with a mental health professional after an incident(s) of unwelcome sexual behavior from a patient?

- ◯ No
- ◯ Yes
- ◯ Unsure
- ◯ I have never experienced such an incident

Have you terminated your relationship with a patient (eg. fired, transferred to another provider) based on unwelcome sexual behaviors?

- ◯ No
- ◯ Yes
- ◯ Unsure
- ◯ I have never experienced such an incident

Please rate the following statements.

I know how to report an incident of unwanted sexual behavior, sexual harassment, or sexual assault by a patient at work.

- ◯ Definitely no
- ◯ Probably no
- ◯ Unsure
- ◯ Probably yes
- ◯ Definitely yes

I know I will be supported if I report an incident of unwanted sexual behavior, sexual harassment, or sexual assault by a patient at work.

- ◯ Definitely no
- ◯ Probably no
- ◯ Unsure
- ◯ Probably yes
- ◯ Definitely yes

Experiencing unwanted sexual behavior, sexual harassment, or sexual assault by a patient has made me feel burned out.

- ◯ Definitely no
- ◯ Probably no
- ◯ Unsure
- ◯ Probably yes
- ◯ Definitely yes
- ◯ Does not apply

Experiencing unwanted sexual behavior, sexual harassment, or sexual assault by a patient has impacted my career path.

- ◯ Definitely no
- ◯ Probably no
- ◯ Unsure
- ◯ Probably yes
- ◯ Definitely yes
- ◯ Does not apply

How has experiencing unwanted sexual behavior at work changed your career?

- ◯ Changed type of practice
- ◯ Changed practice setting
- ◯ Changed specialty
- ◯ Stopped practicing
- ◯ Does not apply
- ◯ Other

Other, please explain: ______________________________

Thank you for taking the time to complete this survey. We welcome any additional comments or insight you have about patient to provider sexual harassment.
